# O “esquecimento global” e os eventos comunicativos extremos

**DOI:** 10.1590/0102-311XPT048825

**Published:** 2025-08-22

**Authors:** Paulo Roberto Vasconcellos-Silva, Luís David Castiel

**Affiliations:** 1 Instituto Oswaldo Cruz, Fundação Oswaldo Cruz, Rio de Janeiro, Brasil.; 2 Escola Nacional de Saúde Pública Sergio Arouca, Fundação Oswaldo Cruz, Rio de Janeiro, Brasil.; 3 Escola de Medicina e Cirurgia, Universidade Federal do Estado do Rio de Janeiro, Rio de Janeiro, Brasil.

**Keywords:** Comunicação em Saúde, Bioética, Desinformação, Hesitação Vacinal, Ciência, Tecnologia e Sociedade, Health Communication, Bioethics, Disinformation, Vaccination Hesitancy, Science, Technology and Society, Comunicación en Salud, Bioética, Desinformación, Vacilación a la Vacunación, Ciencia, Tecnología y Sociedad

## Abstract

No presente ensaio, são descritas as evidências científicas voltadas ao decaimento da memória e do foco pelo hiperestímulo das mídias digitais - aqui apelidado de “esquecimento global”. Argumenta-se que este não se resume a aspectos unicamente psiconeurológicos individuais, mas a processos socioculturais contemporâneos constituídos e alimentados por vetores técnicos, políticos e econômicos convergentes. Discute-se a eticidade da hiperestimulação pelo emprego de potentes dispositivos técnicos de curadoria comunicativa, que resultam em apagamento de referências que constituem a identidade coletiva e facilitam a aceitação de narrativas desinformativas. Nesse contexto, concebemos tal dinâmica de apagamentos e destruição de referências como um processo sociotécnico positivo - e não apenas como mera consequência casual. Usamos o caso emblemático do “rádio do povo” na ascensão do nazismo, mostrando como os eventos comunicativos extremos operam pelo dissenso e oposição de antiversões (negacionismo histórico/científico), estribados em desinformação que incita ao ódio (teorias da conspiração) e simplifica cenários complexos na edificação das pós-verdades. Questiona-se a eticidade das tecnologias digitais contemporâneas que, em paralelo, cultivam os esvaziamentos da memória individual e coletiva pelo “esquecimento global” com o propósito de descaracterizar o passado e paliar psiquicamente a dissonância cognitiva derivada das contradições com o factual. Rejeitamos, assim, a tese do esquecimento fortuito, como reles lacuna ou resíduo consequente a fenômenos psicotécnicos casuais não vinculados. Em síntese, apontamos para a interligação entre o “esquecimento global”, os negacionismos e as pós-verdades - interdependentes em causalidades, propósitos e dinâmicas.

## Abstract (en)

The present essay describes the scientific evidence on the decay of memory and focus caused due to overstimulation by digital media - herein dubbed “global forgetting”. The argument put forth is that this situation is not limited to individual psychoneurological aspects, but rather to contemporary sociocultural processes comprising and fed by convergent technical, political, and economic vectors. This paper addresses the ethicality of overstimulation through the use of powerful technical devices of communicative curation, which result in the erasure of references that constitute collective identity and facilitate the acceptance of disinformation. We conceive these dynamics of erasure and the destruction of references as a positive sociotechnical process - and not merely a casual consequence. We use the emblematic case of the “people’s radio” in the rise of Nazism, showing how extreme communicative events operate through the opposition of anti-versions (historical/scientific denialism) underpinned by disinformation that incites hatred (conspiracy theories) and simplifies complex scenarios in the construction of post-truths. The ethicality of contemporary digital technologies is questioned, which simultaneously cultivate the emptying of individual and collective memory through “global forgetting” with the purpose of rewriting the past and mitigating the cognitive dissonance resulting from contradictions with facts. We thus reject the notion of fortuitous forgetting as a mere gap resulting from unlinked casual psychotechnical phenomena. In summary, we point to the interconnections among “global forgetting”, denialism, and post-truths, which are interdependent in terms of causalities, purposes, and dynamics.

## Resumen

Este ensayo describe la evidencia científica del deterioro de la memoria y la concentración por la hiperestimulación de los medios digitales, aquí nombrado de “olvido global”. Se argumenta que esto no solo se limita a los aspectos psiconeurológicos individuales, sino que también a los procesos socioculturales contemporáneos constituidos y alimentados por vectores técnicos, políticos y económicos convergentes. Se discute la eticidad de la hiperestimulación mediante el uso de potentes dispositivos técnicos de curaduría comunicativa, que ocasionan el borrado de referencias constituyentes de la identidad colectiva y que propician la aceptación de narrativas no informativas. En este contexto, estas dinámicas de borrado y destrucción de referencias se consideran un proceso sociotécnico positivo y no solo como una mera consecuencia casual. Se utiliza el caso emblemático de la “radio popular” en el auge del nazismo para demostrar cómo los eventos comunicativos extremos operan mediante el disenso y la oposición de antiversiones (negacionismo histórico-científico), basadas en la desinformación que incita al odio (teorías conspirativas) y simplifica escenarios complejos en la construcción de posverdades. Se cuestiona la eticidad de las tecnologías digitales contemporáneas que, paralelamente, cultivan el vaciamiento de la memoria individual y colectiva por el “olvido global” con el propósito de caracterizar erróneamente el pasado y atenuar psíquicamente la disonancia cognitiva derivada de las contradicciones con lo fáctico. Por lo tanto, se rechaza la tesis del olvido fortuito como una mera brecha o residuo consecuente a fenómenos psicotécnicos casuales no vinculados. Se concluye al destacar la interconexión entre el “olvido global”, las negaciones y las posverdades, interdependientes en causalidades, propósitos y dinámicas.

## Introdução

A eticidade no uso dos meios de comunicação de massa foi objeto de estudo e questionamento por diversos pensadores ao longo do século XX, sobretudo em face do poder crescente dessas tecnologias em influenciar a opinião pública e moldar percepções coletivas. Autores clássicos como Adorno & Horkheimer, Habermas, Debord e Baudrillard discutiram profundamente as implicações culturais, éticas e políticas das mídias do século XX no que se refere a diversas temáticas envolvendo autonomia, deliberação política e a capacidade crítica dos indivíduos. Na última década, com o advento da internet colaborativa (redes sociais) como expansão da internet distributiva (*websites* e portais de informação), tais questionamentos ampliaram-se em muitos outros terrenos. A eticidade do uso das novas mídias permeia a discussão sobre o uso de dados pessoais de usuários para fins lucrativos sem consentimento ^1^; a influência de algoritmos e sistemas automatizados na perpetuação de desigualdades sociais ^2^ ^,^ ^3^; o uso das tecnologias no reforço ao racismo ^4^; como a promoção da “liberdade na internet” pode ter implicações desastrosas para o futuro da democracia pelo seu emprego por regimes autoritários para vigilância, controle social e manipulação ^5^; a ética (ou sua ausência) da regulação social operada por códigos de software e impactos consequentes ^6^; a distorção de fatos para manipulação antiética da opinião pública com objetivos políticos ^7^; e a desinformação que incita à polarização política como obstáculo ao debate público saudável ^8^. 

Com a ascensão das tecnologias digitais e sua dominância no plano comunicacional, modificou-se a percepção de quem somos e como nos relacionamos com os outros em novas instâncias de relacionamento social, o que nos exige rumos éticos nunca cogitados. Floridi ^9^ ressalta a dependência crescente das tecnologias da informação que acrescenta desafios como a vigilância incessante, a manipulação de dados e o aprofundamento de desigualdades. Argumenta que a quarta revolução (digital) desafia nosso lugar no universo, ressignificando a percepção e compreensão de realidade e identidade. Destaca, assim, a necessidade de uma abordagem ética vigorosa e sofisticada, capaz de lidar com as oportunidades e os desafios complexos, incessantemente dispostos, que advêm de tantas transformações. Bostrom & Yudkowsky ^10^ delineiam um conjunto de critérios éticos que devem ser imperiosamente observados na concepção de algoritmos destinados a interferir ou suplantar o juízo humano: responsabilidade, transparência, auditabilidade, incorruptibilidade e previsibilidade. Entre estes, destaca-se o critério da responsabilidade que deveria merecer consideração prioritária - como base para os demais. Não obstante a tantos aspectos levantados em tantas obras seminais, há pouco material sobre as injunções éticas, sanitárias, políticas, ambientais e educacionais operadas pelos “esquecimentos”.

Cerca de 37 mil pessoas reuniram-se em uma votação pública articulada a análises de lexicógrafos do *Oxford Languages* para escolher um vocábulo ou expressão que representasse os estados de espírito mais influentes que moldaram o ano de 2024. O termo do ano destacado pelo dicionário Oxford foi *brain rot*, definido como uma “*deterioração do estado mental ou intelectual de uma pessoa consequente ao consumo excessivo de conteúdo online considerado trivial ou pouco desafiador*” ^11^. *Brain rot* foi um termo cunhado no século XIX por Henry David Thoreau que, em seu livro *Walden* ^12^, critica inclinações sociais que rejeitam ideias complexas e conceitos que admitem múltiplas interpretações em favor do apego preferencial às imagens simples. Thoreau percebia o fenômeno do apodrecimento cerebral como indício inequívoco de um declínio coletivo - a renúncia ao esforço reflexivo para compreender as coisas da vida dera lugar à debilidade consequente à propensão pelas superficialidades infecundas ^12^. A expressão ganhou recente proeminência por retratar inquietações sobre o impacto do consumo de quantidades excessivas de conteúdo online de baixa qualidade, especialmente nos terrenos das mídias sociais.

O termo *brain rot*, aqui apelidado de “esquecimento global”, não existe como definição formal na literatura acadêmica, embora tenha sido empregado como metáfora que se refere ao declínio nas capacidades cognitivas - particularmente em relação à memória e à atenção - conexo ao uso excessivo de tecnologias digitais ^13^ ^,^ ^14^. A relação entre o *brain rot* e a deterioração na capacidade de memorização globalmente observada, usualmente, é atribuída a diversos fatores ligados aos dispositivos digitais e à fragmentação da atenção causada pela estimulação acelerada e incessante da “multitarefa” (*multitasking*). Como atributo peculiar aos ritmos contemporâneos, o *multitasking* parece comprometer mecanismos de controle executivo e a memória de trabalho - essenciais para a manutenção da atenção sustentada ^15^. Tal impacto de estimulação pelo uso de múltiplas plataformas digitais de comunicação sobrecarrega os sistemas atencionais do cérebro, resultando em limitações na capacidade de manter o foco por períodos prolongados ^16^. A sobrecarga cognitiva decorrente (*cognitive overload*) resulta em uma sociedade de cérebros prejudicados em seu poder de processamento e consolidação de informações e que padece de dificuldades na formação e retenção de referências de longo prazo ^16^ ^,^ ^17^. Em adição, a facilidade de acesso à informação por meio de agendas e mecanismos de busca reduz a necessidade de armazenar dados na memória pessoal - o que ficou conhecido como o *Google effects on memory*. O uso ininterrupto e progressivo de dispositivos para armazenamento de dados triviais faz decair a prática da memorização - desencadeando a “externalização da memória” ^14^ ou “efeito de descarte cognitivo” (*cognitive offloading*), pelo qual o cérebro delega a retenção de informações a artefatos digitais ^14^ ^,^ ^18^. Em outros termos, o uso assíduo de dispositivos eletrônicos e telas de *smartphones* parece estar associado ao declínio da capacidade de memorização por desuso e pelo efeito da fragmentação do foco em prejuízo da consolidação de informações ^16^ ^,^ ^19^. No âmbito educacional, pesquisadores analisaram a interferência de interrupções digitais (notificações e mensagens de texto) no ambiente escolar, concluindo que a presença constante desses estímulos prejudica a consolidação de informações e o desempenho dos estudantes ^20^ ^,^ ^21^. Ademais, estudos neurocientíficos sugerem alterações nos padrões de ativação cerebral durante a busca e o processamento de informações na internet - o que sugere que o uso intensivo das novas mídias pode modificar estratégias nativas de processamento e armazenamento de dados mentais ^13^. Tantas evidências empíricas sustentam a percepção física do *brain rot*, ressaltando a importância de se conquistar equilíbrios éticos entre a utilização das novas tecnologias e a proteção dos processos cognitivos que preservam a memória e o foco em ambientes de constante e intenso estímulo digital.

Ressalta-se que, em conjugação às múltiplas avarias neuropsíquicas peculiares à competitividade que viceja no capitalismo contemporâneo, não são insignificantes os condicionantes extrínsecos na esfera da cultura de massas sob a influência nefasta da “economia da atenção” ^22^. Sob as novas engrenagens do tecnocapitalismo digital, algoritmos colocam-se incessantemente ocupados na microssegmentação de potenciais consumidores para oferta de *fast food* intelectual aos apetites das banalidades. Os *clickbaits* (iscas de cliques) aliciam a atenção do que, não raro, sequer cogitávamos como relevante. Nossas demandas por frivolidades e nosso foco como mercadoria são, assim, oferecidos a anunciantes e plataformas, gerando lucros abundantes e um rastro de desatenção ao que é substantivo em um ambiente cacofônico de abundância informacional. 

De tal forma, perante a tantas convergências eloquentes, no presente texto enfatizam-se aspectos ligados ao decaimento da memória e do foco que transcendem aos aspectos unicamente psiconeurológicos ou individuais. Destacamos uma dimensão estrutural - processos socioculturais constituídos e alimentados por interesses políticos e econômicos em mútua potencialização e com consequências devastadoras. Discute-se, também, a eticidade do comércio do ódio e do apagamento de memórias - sobretudo quando amplificados por potentes dispositivos técnicos de regulação e distorção comunicativa que geram os eventos extremos a que nos referimos no título.

## *Brain rot* e esquecimento global

Há modalidades de *brain rot* talvez pouco noticiadas e muito distintas em causas e consequências do que aqui tratamos: referem-se ao adoecimento coletivo dos moderadores de discursos de ódio postados nas redes sociais.

O Instituto Esalen, localizado em Big Sur, Califórnia, Estados Unidos, coordena um movimento para integração entre ciência, psicologia e espiritualidade, tornando-se um centro para depuração de toxicidades e limpeza espiritual de tecnólogos do Vale do Silício em busca de experiências de transcendência para limpeza espiritual. Moderadores de conteúdo imergem em Esalen na busca pela purgação de males por meio de práticas holísticas de autocuidado, meditação, massagens terapêuticas e *workshops* para autoconhecimento. Esses controladores do fluxo de informações das plataformas digitais lidam diariamente com cargas de trabalho extenuantes de natureza extremamente peculiar. Amparados apenas por manuais volumosos (cada vez mais complexos e pouco precisos), buscam tipificar e deletar os conteúdos indesejáveis, sendo assim persistentemente expostos aos *posts* mais tóxicos e perturbadores ^23^.

Tais deveres os fazem adoecer emocionalmente pelo intenso desgaste inerente à natureza insalubre de seu ofício ^24^ ^,^ ^25^. São forçados à imersão no refugo informacional dos conteúdos espúrios, tensionados entre a competição pela alta produtividade exigida em suas funções e a preservação da saúde mental. Em 2020, o Facebook desembolsou USD 52 milhões no contexto de uma querela judicial movida por moderadores que experimentaram problemas de saúde mental em decorrência de suas funções de “depuração”. O processo judicial foi aberto por Selena Scola, ex-moderadora estadunidense, que descreveu seus ex-colegas como “guardiões de almas” em função da sobrecarga de vídeos sobre decapitações, abusos sexuais e momentos finais da vida de pessoas, entre muitas outras maneiras doentias de se utilizar as redes digitais ^26^.

Na dimensão do cenário global contemporâneo, de acordo com os editores do dicionário Oxford, passamos por um outro processo de franca degradação cognitiva em virtude do “*consumo excessivo de conteúdo online considerado trivial ou pouco desafiador*” ^11^. No entanto, aqui argumentamos que tal degradação atribuída à contínua, incessante e massiva estimulação pode também servir como elemento estruturante de sistemas de poder econômicos e políticos. Assim como qualquer outro sistema de mercado, a economia da atenção baseia-se em relações de trocas em que a atenção humana converte-se em artigo primordial ^27^, sobretudo no panorama da abundância informacional de irrelevâncias. Segundo Caliman ^28^ (p. 11), na lógica da economia da atenção, “*o ater-se a e o ocupar-se com do observador direcionam-se para o que, num movimento de vai e vem, retorna para aquele que vê a possibilidade de ser visto pelo outro e de para ele existir*”. Em outros termos, os elementos centrais à economia da atenção escoram-se na dominância da cultura visual nucleada na visibilidade, aparência e imagem/autoimagem. Trata-se de um modelo econômico que transcende os tradicionais, tendo a atenção e a memorização como recursos valiosos a serem explorados por algoritmos, rolagem infinita e outras estratégias de design persuasivo. Em cenários de sobrecarga de informações, a atenção valoriza-se como um bem escasso e, por conseguinte, principal campo de disputa econômica. Volumosos lucros são extraídos dos estímulos pueris para captura e monetização do foco, assim como dependem, mesmo que indiretamente, dos “esquecimentos”.

Nesse ponto, seria interessante ressaltar que a “pós-verdade” (que simplifica), a desinformação (que engana) e o negacionismo (que rejeita), ao contrário do esquecimento (que apaga), operam positivamente pelo preenchimento de vacuidades, ratificação de crenças e reordenamento de cosmovisões. As *fake news* avançam cotidiana e cumulativamente na direção do convencimento público pelo “varejo do engodo”. A assertividade negacionista constituiu-se como base de um sistema de crenças que, sistematicamente, impõe-se pela rejeição de consensos científicos ou históricos - movidos por propelentes ideológicos, políticos ou religiosos. Por outro lado, o *brain rot* (desconsiderando o apodrecimento emocional dos que submergem profissionalmente nos esgotos da grande rede), ou “esquecimento global”, opera na vacuidade dos “não-registros” de memórias e experiências. Reforçam a imaterialidade do que já é fugaz e dissipa o que já se encontra em rarefação.

Sabe-se que a hiperestimulação midiática sem contrapesos éticos compromete o foco e a fria ponderação coletiva - embaralha rumos e propósitos sociais unicamente para capturar engajamentos (em suas duas conotações). Geram-se manifestações coletivas - justificáveis, tempestivas, proporcionais ou não - sob um efeito de vertigem, sensação de vazio de referências e aceleração de tempo (perdido) que incita a atenção para explicações de mundo simplórias, em retroalimentação. Tais simplificações convenientemente envolvem conspirações e ameaças do “eles” contra o “nós”, sabido que esses conteúdos revertem-se em engajamentos e lucros que perduram. Assim, se as informações falsas são tijolos na construção dos muros dos negacionismos e da pós-verdade, o “esquecimento global” seria a argamassa - a penúria da memória e da capacidade de absorver e entronizar conhecimentos faz abdicar a percepção e reflexão sobre as coisas do mundo.

## Eventos comunicativos extremos

Maquinações que produzem artefatos para distorção da comunicação pública avançam contra a proposição e implementação de intervenções de relevância sanitária, política e ambiental - são eventos comunicativos extremos na noosfera. Tais eventos, engrenados como discursos perlocucionários voltados ao discernimento público, podem matar e fazer adoecer. A desinformação, o negacionismo e a pós-verdade, como abalos ou rupturas, fazem estremecer bases epistêmicas do *Homo sapiens* há tempos. Há exemplos próximos bem conhecidos como os “Protocolos dos Sábios do Sião”, o discurso pró tabagista e a ideia da “superioridade da raça ariana” que acenderam e fizeram ascender o nazismo. São abundantes no discurso dos líderes populistas e promovem dilúvios de águas turvas pelos fluxos da “hesitação vacinal”, do “terraplanismo” e dos discursos antiambientalistas, entre outros. Atuam pela desinformação das teorias conspiratórias consolidada em pós-verdades constituídas por negacionismos em mútua potencialização com as deslembranças.

### Disseminação da mentira pelas falsas narrativas 

Apesar do que se faz parecer, as falsas narrativas, falsas notícias ou *fake news* não têm início na dimensão das mídias digitais ou tradicionais, nem sequer representam a juventude de fatos informacionais na noosfera. Mentiras são divulgadas para enganar desde sempre, nos mais variados meios sociais e em todas as épocas históricas. Rumores e fofocas são produto da interação social e, em sua geração e propagação, não dependem de artefatos ou ambientes digitais - são construções sociais decorrentes da inter-relação comunicativa entre grupos com propósito de estabelecer ou reforçar alianças. Segundo os teóricos no campo, na medida em que grupos de hominídeos falantes expandiu-se, as interações face a face e o *social grooming* (cuidado físico e social) tornaram-se insuficientes como fator de coesão social. Nesse ponto evolucionário, os mitos e cosmologias, assim como os boatos e as fofocas, passaram a potencializar as ligações sociais, gerando crenças e costumes aceitáveis/inapropriados; estabelecendo ordenamentos e hierarquias; aproximando aliados e rejeitando inimigos; separando o sagrado do profano.

Em síntese, idealizações míticas ou simples mentiras colocaram-se como vetores de conexão social por meio das fronteiras narrativas a separar o aceitável do condenável no comportamento tribal ^29^ ^,^ ^30^ ^,^ ^31^. Ainda hoje as narrativas servem à consolidação de identidades de grupo - independentemente da veracidade das informações compartilhadas. Separam “nós” de “eles” a reforçar fronteiras simbólicas entre nichos sociais, políticos ou étnicos. Como propelente maior das teorias da conspiração, possuem o poder de idealizar um “outro” contra o qual o grupo se mobiliza, estimulando percepção de solidariedade interna em contraste com a desconfiança externa ^32^ ^,^ ^33^.

Nos primórdios da era digital, acreditávamos nas mudanças positivas que certamente adviriam do estado de hiperconexão global e acessibilidade irrestrita a um clique ou deslize de dedos. Pierre Lévy expressou seu vívido otimismo sobre a nova cibercultura ^34^ - espaço de construção para vindoura inteligência coletiva ^35^ e educação libertária para a iminente ciberdemocracia ^36^.

### Onde foi que erramos?

Tal visão idealizada foi substituída em poucos anos pelo reconhecimento de que o ecossistema de informações - nossa noosfera - encontra-se perigosamente poluída e insalubre, a nos dividir em bolhas em acelerada marcha de involução. Indivíduos que chafurdam em “tocas de coelho” não mais se importam com a veracidade das teorias repassadas ou mesmo com a justiça de suas crenças motoras - contentam-se em “estar certos” para perpetuar os ciclos de viralização entre seus pares ^23^ ^,^ ^32^. Como escravos de seus vieses de confirmação, compartilham falsas narrativas sem verificar origens, refletir sobre consequências ou analisar a consistência interna dessas partículas conspiratórias.

As falsas narrativas sofrem mutações e evoluem, aninhando-se, adaptando-se e, não raro, fortalecendo-se a partir de discursos hospedeiros. Como blocos das teorias da conspiração e pós-verdade, adquirem forma e mecanismo de perpetração análoga à dos vírus - dotados de capacidade de replicação, embora utilizando o maquinário do organismo “infectado”. Como partículas discursivas, não contam com metabolismo próprio, raramente possuem um *logos* estruturante independente e, quando isoladas, são pouco úteis às extrapolações universais. Assim como os boatos, se não conseguem abrigo em um lócus de contextura narrativa convergente, perdem sentido, força e valor ^37^. Retiram boa parte de suas energias de viralização do *pathos* odioso, gerado por ressentimentos e frustrações de indivíduos que se consideram expropriados, excluídos ou irrelevantes. Assim como os vírus, são, portanto, parasitas obrigatórios - não sobrevivem fora das câmaras de eco e do sistema epistêmico enviesado pelos filtros de bolha ^32^. Não se replicam fora da invasão pelo engano e dependem do suporte de uma mistificação estruturante prévia.

### O negacionismo e as conspirações 

O negacionismo manifesta-se de variadas formas, como a negação de eventos históricos comprovados ou a rejeição de teorias científicas estabelecidas. Rejeitam qualquer diálogo que tragam críticas pertinentes, evidências empíricas, argumentos lógicos, as premissas de um debate público racional. Os negacionismos guardariam diversos traços de parentesco entre si que, em conjunto, representariam um dos maiores e mais complexos desafios à frente das sociedades contemporâneas ^23^. No atual momento de extremos, nossa compreensão sobre tais fenômenos talvez se amplie ao conceber o “esquecimento global”, o negacionismo e a pós-verdade como uma matriz de fenômenos desencadeados pelas novas mídias em mútua alimentação e contexto histórico especialmente propício.

Desde meados do último século, houve expressiva proliferação de negacionismos científicos ligados à saúde, ora afirmando a existência de fatos não verificáveis, ora produzindo reinterpretações com maior ou menor grau de plausibilidade, mas, quase sempre, investindo contra a hegemonia da ciência na afirmação do real. Como exemplo de negacionismo corporativo, a indústria do tabaco financiou cientistas, ignorou evidências robustas e promoveu campanhas de desinformação para negar a relação entre o tabagismo e diversas doenças. Mesmo perante evidências esmagadoras, a indústria valeu-se de todas as táticas disponíveis para reafirmar tal negação, evitar condenação em processos e regulamentações mais rígidas ^38^. Na década de 1980, com a disseminação da pandemia de HIV/aids, surgiram teorias que negavam a existência da doença ou desacreditavam o papel do vírus na síndrome, como desinformação publicizada pelo cientista Peter Duesberg, o que comprometeu políticas públicas em alguns países, resultando em milhares de mortes evitáveis ^38^ ^,^ ^39^.

No final do século XX, um dos casos mais proeminentes de negacionismo em termos de saúde pública foi representado pelo movimento antivacina, amplificado pelo artigo fraudulento do Dr. Andrew Wakefield, que ligava enganosamente a vacina tríplice viral ao autismo ^40^ ^,^ ^41^. Até hoje, a hesitação vacinal tem exercido impactos significativos na saúde pública global - no Brasil, tem contribuído significativamente para a redução da cobertura vacinal e reemergência de doenças imunopreveníveis. A vacina contra o sarampo (ou tríplice viral difamada por Wakefield) teve cobertura nacional de 67,3% em 2021 - queda drástica se comparada aos níveis acima de 90% nas décadas anteriores ^42^. Assim, uma teoria negacionista articulada a um movimento crescente no terreno político e cultural - amparado até por líderes políticos proeminentes ^43^ - tem contribuído para o aumento de surtos de doenças previamente controladas ou eliminadas. Logo após a decretação do estado de pandemia da COVID-19, houve desenfreada proliferação de teorias conspiratórias que negavam a gravidade da doença e a eficácia das vacinas, além de promover terapias curativas de comprovação (à época) questionável ^44^. Pais que acreditavam estar protegendo seus filhos buscaram falsa segurança nas narrativas antivacina - em forte dissonância cognitiva com informações amparada pelas evidências científicas sobre a eficácia e segurança dos imunizantes.

A aglutinação e proliferação destas, entre tantas outras, tendências anticientíficas se dá em ambientes comunicacionais isolados, como bolhas. Também conhecidas como “câmaras de eco”, constituem-se na noosfera como um ecossistema de informação enviesada, permanentemente ocupado em produzir e repercutir crenças tão absurdas quanto inarredáveis ^32^ ^,^ ^44^ ^,^ ^45^. No mundo exterior, a mera refutação com argumentos factuais é recebida com antagonismo visceral. As premissas de um debate público racional, permeado por argumentação lógica em condições comunicativamente livres e equilibradas, não são sequer cogitadas. Nesses territórios áridos da noosfera, não há dissensos e a diversidade de pensamentos é inexistente - o que cria espaços para versões cada vez mais radicais em mútua alimentação. Esses nichos são nutridos por teorias da conspiração negacionistas produzidas e disseminadas pelas redes de bots programados para compartilhar e redistribuir conteúdo conspiratório.

Os *bots* operam na visibilidade de conteúdos, independentemente de sua veracidade. Contribuem para a perpetração e reafirmação de “truísmos”, sobretudo nas plataformas que priorizam engajamento algorítmico e visibilidade pública em tempo real (Twitter/X, Facebook, Instagram, YouTube e, mais recentemente, TikTok). Podem ser programados para reagir a conteúdos específicos de forma massiva, induzindo os algoritmos de redes sociais a interpretar tais interações como indícios de relevância. Podem produzir uma ilusão de consenso (*astroturfing*) e reforçar as câmaras de eco, atuando em bolhas e ambientes ideologicamente homogêneos. Em síntese, são produtos aprimorados para semear dúvidas e desconfiança sobre evidências científicas, o que produz efeito de “diluição” da informação qualificada. Sua potência de alcance varia de acordo com o design da plataforma, as políticas de moderação adotadas e o nível de acesso facultado a agentes externos (humanos ou automatizados) para “raspar” (*scraping*), analisar e interferir com dados da plataforma ^1^ ^,^ ^23^ ^,^ ^46^ ^,^ ^47^ ^,^ ^48^. Grupos financiados por indústrias de combustíveis fósseis e *think tanks* conservadores têm produzido desinformação direcionada a comunidades céticas ou já predispostas a aceitar e divulgar desinformação ambiental. O negacionismo climático configura-se a partir de “evidências” que comprovam que as mudanças no clima percebidas constituem uma “farsa” composta por evidências manipuladas por cientistas financiados ou alinhados a agendas políticas.

Atualmente os negacionismos erguem-se e estruturam-se como categorias discursivas de pós-verdade por meio de falsas narrativas veiculadas por boatos, opiniões dolosas ou culposas de figuras proeminentes (ou não) nas redes sociais digitais ^49^. Como narrativas desinformativas, consolidam-se como eventos comunicativos extremos, produzidos à base de matéria conspiratória; alusão a medos e ameaças iminentes; constituição lógica rudimentar; e nexos causais pouco complexos, como vetores essenciais à eficiência da viralização. Constituem-se como teias de crenças que, sistematicamente e em sincronia, negam consensos histórico-científicos e evidências empíricas.

Fisher ^23^ descreveu em detalhes as portas de entrada, percursos de radicalização e terríveis consequências originadas por milhões de incautos atraídos por ideias negacionistas que submergem nas “tocas de coelho”. Estas podem ser definidas como conteúdos conspiratórios ou negacionistas potencializados por algoritmos das plataformas de mídias sociais que trabalham na captação de novos adeptos e seu engajamento. Esses algoritmos, norteados por métricas que objetivam “tempo de tela”, priorizam os conteúdos mais extremados - considerados os mais eficazes para conquista de radicalização crescente e engajamento derivado que, em casos frequentes, conduzem a comportamentos obsessivos de buscas ^23^ e sequelas mentais por dissonância cognitiva.

### As edificações da pós-verdade

Em 2016, logo após o referendo do Brexit e a escolha de Donald Trump nas eleições presidenciais estadunidenses, um outro termo implicado nas torceduras epistêmicas massivas de nosso tempo, “pós-verdade” (*post-truth*), foi designado pelo dicionário Oxford como palavra de destaque do ano. O termo foi concebido em 1992 no contexto histórico da Guerra do Golfo e da monumental falsa narrativa institucional estadunidense que a engendrou. Em época bem anterior à criação e popularização das redes sociais digitais, Steve Tesich referia-se ao fato de que, numa sociedade dominada pela pós-verdade, os fatos importam menos do que as crenças ^45^. Interpretações sobre um determinado fato teriam mais importância do que o próprio fato em si, reafirmando a precedência de versões e narrativas paralelas sobre o sentido e propósito da “verdade”, o que implica sua desvalorização por superação.

As novas tecnologias e as redes digitais desencadearam transformações qualitativas ao processo de comunicação pública em contraste com a dilatação quantitativa proporcionada pelos meios de comunicação tradicionais. O rádio, a televisão e os jornais a todos alcançavam em seus formatos, dinâmicas e peculiares modalidades de penetração, acenando com a veracidade/objetividade de fatos pré-selecionados e interpretações convenientes às agendas das elites. No contexto atual, a pós-verdade gerada pelas falsas narrativas adentra como estreante que, de forma estridente e não raro desonesta ^50^, insurge-se pela via da dissonância cognitiva e negação ostensiva de objetos factuais. Interessante mencionar Keyes ^50^, que explora as raízes do fenômeno “pós-verdade”, argumentando que o cenário contemporâneo é caracterizado por uma aceitação ampliada e dócil de falsidades e enganos, fenômeno intensificado pelas novas tecnologias e mídias sociais. Nos Estados Unidos e no Reino Unido, o ano de 2016 foi marcado por debates políticos regidos pela dominância de afirmações sem substrato empírico que, apesar de falaciosas, instrumentalizavam temores, inseguranças e a autopercepção de irrelevância política entre parcelas expressivas da sociedade. Narrativas alternativas substituíram a consubstanciação do real por versões cinzeladas por mentiras ostensivas e apelos emocionais ao medo e ao ódio constituinte da intolerância. A “verdade” fora eclipsada pelas versões pessoais, polarizações ideológicas e manipulações midiáticas obstinadas em priorizar conspirações.

As novas dinâmicas e regimes de aceleração das mídias digitais alavancaram a desinformação pela produção de filtros e bolhas de desinformação a obliterar qualquer perspectiva de consenso fundamentado em verdades factuais. De forma geral, a erosão da verdade tem se dado pela abertura de amplas avenidas para o negacionismo científico, as falsas narrativas e os balizamentos oblíquos da pós-verdade que prosperam perante interpretações pessoais regidas por dissonância cognitiva (usualmente de gênese conspiratória). Importante ressaltar que não há linearidade direta entre o conceito de pós-verdade e engodo/mentira (embora guarde relação estreita com ideologias), nem paralelos como o caráter doloso/culposo das falsas narrativas ^51^. Como ferramenta essencial às ideologias, a pós-verdade também presta-se à deformação da percepção das relações sociais, naturalizando desigualdades, legitimando o poder de classes dominantes ou produzindo alinhamento a falsas consciências ^52^, entre diversas outras formas de emprego com propósitos comerciais, políticos ou morais. Guarda como propósito central implantar sentimentos e percepções que se aprofundam em intransigência e sectarismo a favorecer interesses grupais de ordem política, econômica ou cultural para os quais pouco importa para a veracidade dos fatos ^53^. Acerca de sua ligação atávica com as ideologias, Hannah Arendt não se debruçou especificamente sobre o tema, embora tenha levantado aspectos intimamente relacionados à manipulação da verdade em seu livro *Entre o Passado e o Futuro* ^54^, no qual discorre sobre como os fatos podem ser distorcidos por sistemas políticos para alcançar objetivos. Sua análise antecipou debates mais recentes sobre o valor da veracidade em uma era de desinformação.

A pós-verdade germina e prospera por negligências (ou interdições relativas) à pesquisa - conjugadas ou não a diversos vetores - para ocultar ou menosprezar fatos históricos; pela desconfiança/desprezo nas autoridades oficiais; para conjurar inimigos; pela rejeição persecutória a verdades tradicionalmente estabelecidas ^49^ ^,^ ^50^ ^,^ ^55^. Importante acrescentar que o “império do ódio” em que submergiram milhões pela força do embate visceral entre cosmovisões opostas - mal informadas ou não - comprometeu a viabilidade do discernimento necessário a qualquer tipo de reflexão crítica aprofundada ^32^. Sob perspectivas pós-modernas, todas as certezas consolidadas como princípios, normas e práticas passaram a ser questionadas ou criticadas. Como visto ao longo da pandemia de COVID-19, o mesmo aconteceu com as “certezas científicas”. O *logos* da ciência esvaziou-se perante o *ethos* de tantas autoridades em destaque e ao *pathos* do medo e do sofrimento coletivo em cacofonia ^44^. Assim, a micropulverização e descentralização de enunciadores e receptores de informação pelas novas tecnologias, conjugadas ao pânico frente à iminência da morte, geraram consequências desastrosas em óbitos e grave adoecimento. Diante de graves narrativas de riscos, o sono da razão comunicativa instalou-se, gerando alguns monstros ^56^.

## A natureza estrutural e positiva do “esquecimento global”

Pela ótica freudiana, lapsos de memórias podem expressar mecanismos defensivos de repressão psíquica para lidar com conflitos, pensamentos ou lembranças desconfortáveis, usualmente de natureza sexual ou agressiva. A deslembrança de conteúdos interditos protegeria a estabilidade psíquica do sujeito de elementos perturbadores ^57^. Lacan ^58^, sob uma reinterpretação estruturalista, descreve o inconsciente constituído como linguagem e retrata os lapsos como rupturas no encadeamento significante na sequência de signos que constituem a subjetividade. Winnicott ^59^ se vale de outras perspectivas ao analisar o esquecimento à luz das relações objetais e do desenvolvimento emocional. Sugere que a deslembrança pode estar ligada a falhas em experiências precoces de cuidado e acolhimento. Nas insuficiências ambientais em sustentar o sujeito em momentos de vulnerabilidade, ocorrem falhas no processo de simbolização e de integração psíquica - vivências não suficientemente internalizadas de forma segura surgem como lacunas no processo de amadurecimento emocional ^59^.

Em síntese, a psicanálise em múltiplas vertentes percebe lapsos de memória como expressões negativas, ou “vazios” em uma dinâmica psíquica complexa, envolvendo a repressão, o desejo inconsciente ou falhas na simbolização. Tanto Freud (enfatizando o papel da repressão) como Lacan (destacando a ruptura no registro simbólico) e Winnicott (abordando deficiências nas relações objetais) adotam perspectivas negativas quanto aos lapsos de memória - sobretudo na ênfase às exclusões de registros, valorizando o que o fenômeno reprime, faz omitir ou evanescer. Nietzsche acrescentou alguma substância aos vazios da memória ao descrever o esquecimento como uma necessidade existencial, através da qual é possível retomar a vida livre de débitos e consequentes ressentimentos ^60^. Em suas palavras, “*em meio à menor como em meio à maior felicidade é sempre uma única coisa que torna a felicidade o que ela é: o poder-esquecer*” ^61^ (p. 9). Relevante destacar a ironia no tensionamento filosófico entre a perspectiva de Nietzsche e a crítica que permeia o presente texto - enquanto aquela refere-se à subjetividade, à “virtude do esquecer”, esta refere-se ao coletivo, à expropriação de referências para fragilização cognitiva de um povo. A primeira está ligada ao poder libertador do perdão e à paliação de ressentimentos autoimpostos - a última é recurso à dominação. Para um indivíduo, o esquecimento pode ser um apagamento ativo de reveses, como um desapego saudável. Mas também, ao nível social, pode estar a serviço de um mecanismo de espoliação de ícones que constituem a percepção crítica de uma sociedade.

Não obstante às ponderações psicanalíticas/existenciais, pergunta-se: nos planos coletivos e no contexto da ordem tecnopolítica vigente, essas deslembranças poderiam representar atributos positivos em sua gênese, reprodução e propósitos? Lacunas e vazios poderiam adquirir substância ontológica, como matéria fundamental necessária ao entendimento de novos regimes de dominação econômica/política? Talvez tais interrogações, conduzidas como questão filosófica, sirvam à compreensão de dimensões transcendentes ao conceito de mero desaparecimento de lembranças pela incessante exposição às telas. A necessidade de substância ontológica inerente a determinados conceitos desempenha um papel essencial em sua fundamentação filosófica e na construção do entendimento acerca de panoramas complexos. Fornece bases para a compreensão de como artefatos e entidades mantêm-se coesos e contínuos em suas transformações e relações com outros elementos - aparentemente desconexos. A substância como aquilo que “existe por si” colabora na formulação de noções que distinguem melhor o essencial do acidental, o casual do causal. Estabelece, mesmo que de forma provisória, uma estrutura conceitual estável, útil a descrever uma realidade complexa ^62^.

Pertinente acrescentar que há substância assertiva aplicada a conceitos essencialmente positivos como “desinformação”, “pós-verdade” e até mesmo na concepção de “negacionismo” (apesar de sua definição que ressalta uma rejeição do consagrado). Assim colocado, haveria conexão entre os esquecimentos globais e a ampliação/perpetuação dos estados de degradação epistêmica ou paliação das dissonâncias cognitivas? Seria o esquecimento e a falta de foco convenientes aos interesses espúrios que mobilizam incautos insatisfeitos em desconexão de causas e penúria de referências essenciais na direção de pautas indistintas? Câmaras de eco, bolhas de filtros e tocas de coelho, potencializadas pela cacofonia dos novos canais de comunicação, guardariam alguma relação simbiótica com a evanescência das lembranças que deveriam fornecer matéria ao pensamento crítico? O exílio epistêmico autoimposto por meio da curadoria de algoritmos e bolhas comunicativas dependeria em sua gênese e desenvolvimento de alguma sorte de obliteração de memórias? Seria esta útil às distorções que nublassem o discernimento e a percepção do real? Tais extermínios da memória abririam sítios férteis para os negacionismos e narrativas falaciosas? Como paliar a dissonância cognitiva sobre evidências senão esquecendo-as?

No sentido dessa discussão, seria interessante analisar a emergência da ideologia nazista na Alemanha como um sistema de manipulação da memória histórica e sistemático apagamento de eventos e narrativas da consciência coletiva alemã. Hitler e Goebbels, arquitetos das maquinações implicadas na narrativa nacional que exterminaria as demais, abusavam das eliminações, supressões e distorções históricas não alinhadas à ideologia que se imporia pela destruição. Nesse processo de difusão mentiras e apagamentos, é relevante mencionar o poder de um artefato tecnológico sumamente influente à época - um suporte essencial ao maquinário nazista que raramente é mencionado. Interessante notar que seu emprego em larga escala no território alemão guarda preocupantes parentescos com o contexto tecnocomunicativo do século XXI. Um dispositivo (em sua conotação de artefato físico) de difusão foi empregado incessantemente pela propaganda nazista desde 1933, quando Hitler ascendeu ao poder. Um elegante gabinete de baquelite envolvia um aparelho desenvolvido pelo engenheiro Otto Griessing, a pedido de Joseph Goebbels, como um modelo simplificado de rádio capaz de receber emissões a curtas distâncias. O modelo VE301 era vendido a cerca de 20% de seu valor de mercado e fora subvencionado pelo governo com o intento de ser um *Volksempfänger* (rádio do povo), desenvolvido para repercutir música folclórica, de início. Pouco a pouco, a programação do artefato passou a ser ocupada pelas falas do Führer, notícias manipuladas sobre política internacional e sobre a “*grave ameaça dos judeus*” ^64^. Nas palavras de Goebbels: “*Não fazemos segredo disso: o rádio é nosso e de mais ninguém. E vamos colocar o rádio a serviço da nossa ideologia*” ^63^. Por tais meios e propósitos, acelerou-se o engendramento da narrativa nazista e seus recursos às supressões, distorções e negacionismos sistemáticos que culminaram na completa obliteração da memória coletiva sobre a longa e rica história da contribuição dos judeus para a sociedade alemã ^64^ ^,^ ^65^. No decorrer do Terceiro Reich, as referências de destaque positivo sobre os judeus na economia, ciência, arte e literatura alemã foram sendo apagadas, o que culminou em sua completa desumanização em nome da “pureza racial” e “estabilidade do estado alemão” ^66^. Entraram em ação todas as táticas necessárias à omissão deliberada de seu papel histórico, em paralelo à manipulação ostensiva da imagem do grupo como uma ameaça à economia, à saúde e à sobrevivência do povo ariano ^64^.

Observa-se assim a potência de recursos tecnológicos de propagação de mentiras, amplificando a substituição de fatos por imagens caluniosas e articulada ao sistemático apagamento de referências históricas.

O “nós”, sob ameaça, contra o “eles”, como raízes de todos os infortúnios, necessitava da negação de um papel histórico relevante de um segmento da sociedade e, no limite, do apagamento dos rastros de sua humanidade.

Assertivas ostensivas conjugadas a apagamentos sistemáticos pelos recursos radiofônicos conduziram à aceitação das leis antissemitas que geraram as condições para o Holocausto. Convém sempre lembrar que, em nosso país, também experimentamos um período de esforços para deterioração deliberada da memória coletiva no que se refere à tentativa de substituição da ideia de Golpe Militar de 1964 pela versão de “movimento popular liderado por militares no combate ao comunismo”. 

A dissonância cognitiva desencadeada pela rejeição à vacina necessitou do apagamento das memórias das campanhas de vacinação de nossas infâncias protagonizadas pelo “Zé Gotinha”. Para negacionistas, o aquecimento do planeta, os eventos climáticos extremos e desastres naturais consequentes, cada vez mais presentes e frequentes, exigem o apagamento da percepção física do clima. Há diversos exemplos contemporâneos, distribuídos por diversas outras partes do globo, sobre pós-verdades odiosas disseminadas pelas novas mídias em conjugação com o apagamento de memórias. De forma análoga ao *Volksempfänger*, o Facebook tem contribuído para amplificação de discursos de ódio como combustível para limpezas étnicas em ambientes políticos de democracias frágeis em Mianmar ^67^, no Sri Lanka ^68^ e na Etiópia ^69^. Em resumo, o que se deseja aqui enfatizar é a eticidade do emprego do ódio e apagamento de memórias - sobretudo quando amplificados por potentes dispositivos de regulação comunicativa oferecidos a preços acessíveis a populações economicamente depauperadas em que se prenuncia climas tormentosos de limpeza étnica ^33^ ^,^ ^70^.

## Conclusão - o mundo dos átomos e o “não lugar” atemporal dos fluxos de elétrons

Em síntese, aqui nos referimos ao esquecimento global e sua versão anglofônica (*brain rot*) como a precarização de três elementos cognitivos - memória, foco e senso crítico - gerada em duas dimensões interconectadas em mútua potencialização: como expropriação coletiva implicada na economia da atenção; e como sequela neuropsíquica individual pela externalização da memória e as sobrecargas derivadas da competitividade (*overload*, *multitasking*). Talvez fosse possível incluir o apodrecimento mental dos moderadores de conteúdos (expostos aos refugos do ódio e das perversões humanas) como insalubridade na dimensão laboral - o que não caberia discutir aqui em virtude dos formatos editoriais disponíveis.

Nicholas Negroponte ^71^, ao abordar a transição da sociedade analógica para a era digital, introduziu o conceito de “mundo dos átomos” e “mundo dos elétrons” para ilustrar diferenças em suas perspectivas sobre o mundo físico tangível e o digital que o substituiria. O mundo dos átomos, no qual evoluímos desde as cavernas, é limitado pelo tempo e espaço e refere-se ao universo sensível, povoado por objetos tangíveis: livros e jornais de papel, obras de arte, edificações, veículos e demais objetos dotados de massa e presença. Por outro lado, o mundo dos elétrons representa o universo digital, onde a informação é transmitida e processada de forma imaterial e instantânea. Elementos do mundo dos átomos podem ser convertidos em fluxos de elétrons com fins de representação, adquirindo a forma de som, imagens, texto etc. - o que permite a superação de fronteiras geográficas pela facilidade de sua transmissão em velocidade quase instantânea.

O conceito de Negroponte ^71^ fazia todo sentido no contexto da “era da inocência” da internet e pode ser útil, em parte, na presente discussão. Vivemos em uma era de progressiva transição da “memória dos átomos” - constituída pelas fotografias, jornais e revistas em papel - para a relativa vacuidade inerente à “memória dos fluxos de elétrons”, das fotografias esquecidas nos *smartphones*, dos apelos imagéticos do Instagram, do YouTube e do TikTok. Interessante notar que nos acostumamos a abandonar a materialidade da memória de viagens, passeios, momentos e comemorações felizes. Importante perceber que objetos físicos são dotados de presença - deixam resíduos sensíveis pelo tato, textura, peso, temperatura, odor e até por sua fragilidade. Tudo isso foi confinado aos fluxos de elétrons nos “não-lugares” do Instagram ou apenas esquecido nos *smartphones* - não mais nos álbuns de fotos da estante da sala. As páginas de jornais de papel, relidas quase por acidente ao forrar o chão para os animais de estimação ou embrulhar objetos frágeis, também se foram. O que perderemos com isso? Daqui a um milênio, o que arqueólogos e historiadores encontrarão como rastros de nosso tempo? Tuítes de celebridades? Imagens no Instagram? Vídeos do YouTube ou do TikTok?

A penúria de referências críticas e atenção precária ao que é relevante no mundo dos átomos traz sérias consequências. O esquecimento global e a falta de conexão com o real abrem amplos espaços para distrações incessantes, em rolagem infinita. O *Volksempfänger* foi o arauto de uma era de desequilíbrios - dilúvios incessantes de elétrons dão forma, veículo e poder de multiplicação aos discursos de ódio; à linguagem da violência; ao politicamente incorreto; às teorias conspiratórias; aos risos perante o abjeto; ao terraplanismo; à hesitação vacinal; à mitologia sobre as agendas globalistas de dominação; às tocas de coelho; aos *Pizzagates*; aos *Gamergates*; aos *4chans*; e aos líderes populistas que atualmente corrompem a democracia.

Ou seria tudo isso apenas uma teoria da conspiração?
